# Study on the regioselectivity of the N-ethylation reaction of *N*-benzyl-4-oxo-1,4-dihydroquinoline-3-carboxamide

**DOI:** 10.3762/bjoc.15.35

**Published:** 2019-02-12

**Authors:** Pedro N Batalha, Luana da S M Forezi, Maria Clara R Freitas, Nathalia M de C Tolentino, Ednilsom Orestes, José Walkimar de M Carneiro, Fernanda da C S Boechat, Maria Cecília B V de Souza

**Affiliations:** 1 Instituto de Química, Universidade Federal Fluminense, Niterói, 24020-150, Brazil; 2 Instituto de Física, LDRX-UFF, Universidade Federal Fluminense Niterói, 24210-347, Brazil; 3 Departamento de Química, Pavilhão de Química, Universidade Federal Rural do Rio de Janeiro, Seropédica, Seropédica, RJ, 23890-000, Brazil; 4 Escola de Engenharia Industrial Metalúrgica, Universidade Federal Fluminense, Volta Redonda, 27255-125, Brazil

**Keywords:** alkylation, carboxamide, oxoquinoline, quinolone, regioselectivity

## Abstract

4-Oxoquinolines are a class of organic substances of great importance in medicinal chemistry, due to their biological and synthetic versatility. *N*-1-Alkylated-4-oxoquinoline derivatives have been associated with different pharmacological activities such as antibacterial and antiviral. The presence of a carboxamide unit connected to carbon C-3 of the 4-oxoquinoline core has been associated with various biological activities. Experimentally, the N-ethylation reaction of *N*-benzyl-4-oxo-1,4-dihydroquinoline-3-carboxamide occurs at the nitrogen of the oxoquinoline group, in a regiosselective way. In this work, we employed DFT methods to investigate the regiosselective ethylation reaction of *N*-benzyl-4-oxo-1,4-dihydroquinoline-3-carboxamide, evaluating its acid/base behavior and possible reaction paths.

## Introduction

**Figure 1:**
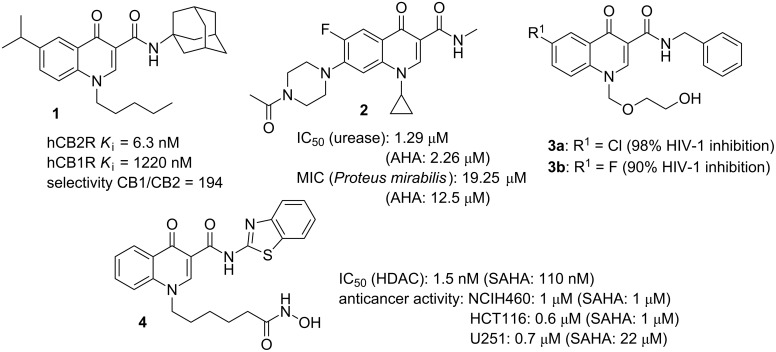
Structures of some bioactive 4-oxoquinoline-3-carboxamide derivatives **1**–**4** with different bioactive profiles. *K*_i_ = binding affinity; AHA = acetohydroxamic acid (standard urease inhibitor); SAHA = suberoylanilide hydroxamic acid (an FDA approved drug for cutaneous T-cell lymphoma); NCIH460 = lung cancer cell line; HCT116 = colon cancer cell line; U251 = glioma cell line.

Since the discovery of the antibacterial agent nalidixic acid, as a byproduct from the synthesis of chloroquine, the medicinal interest in 4-oxoquinolines as bioactive substances has exponentially grown over the years. Nowadays, some of the most important antibiotics used in the treatment of bacterial infections are 4-oxoquinoline derivatives, namely, ciprofloxacin, levofloxacin, lomefloxacin and others [1–2]. Even though the antibacterial profile has been the most common bioactivity associated with this class of substances [1], other types of pharmacological activities have also been explored and were successfully described by researchers around the world [3–4], such as antiviral [5–6], antiplasmoidal [7–8], anticancer [9–10], and trypanocide [11] activities. 4-Oxoquinoline-3-carboxamide derivatives, more specifically, have shown to be a promising structural scaffold for pharmacological profiles [12–22]. For example, Pasquini and co-workers have studied the application of *N*-adamantyl-4-oxoquinoline-3-carboxamide derivative **1** as selective cannabinoid type 2 receptor ligand with agonistic effect for analgesic response [13]. Abdullah and collaborators described the synthesis and investigation of the bacteria urease inhibitory activity for ciprofloxacin derivatives, including the amide **2**, which presented a remarkable IC_50_ value for urease inhibition and was capable of inhibiting *Proteus mirabilis* growth [14]. As another example, in a previous work we described the synthesis and antiviral activity of some 4-oxoquinoline acyclonucleosides **3a** and **3b** [15] and studies on their anticancer activity are also underway. It is also worth mentioning that derivative **4** presented an excellent inhibitory profile for the enzyme hystone deacetylase (HDAC), and anticancer activity for three cancer cell lines (Figure 1) [16].

Although it is not a general rule for achieving a bioactive profile, any groups attached to C-3 of the 4-oxoquinoline moiety, especially those containing a hydrogen bond donor group, such as a carboxyl, an acyl hydrazide or a carboxamide group, may contribute to enhance the bioactivity. This fact could be explained by the coplanarity induced by the C-4 carbonyl hydrogen bond interactions with biological targets [3] or complexation with physiological metal cations such as magnesium and zinc [23].

**Figure 2:**
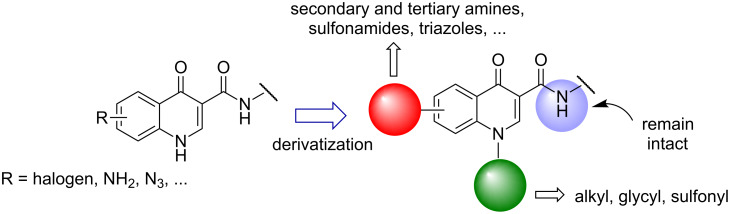
Structural modifications on the 4-oxo-1,4-dihydroquinoline-3-carboxamide scaffold.

Besides the derivatives **3a** and **3b** mentioned above, we have been putting some effort on synthesizing different 4-oxoquinoline-3-carboxamide derivatives as potential anticancer agents [13]. The synthesis of such derivatives have been planned considering that, once having obtained the 4-oxo-1,4-dihydroquinoline-3-carboxamide scaffold, accomplished through temperatures above 200 °C, any derivatization afterwards should maintain the N–H carboxamide group intact, in order to provide the hydrogen bond donor group attached to C-3, which is usually related to the bioactivity of such compounds (Figure 2) [3,23].

In order to obtain an *N*1-alkylated-4-oxoquinoline derivative, the aliphatic nucleophilic substitution reaction can be employed, in which the 4-oxoquinoline nucleus acts as an azanucleophile, reacting with different alkyl halides. This reaction leads to products with high yields, and no byproducts are isolated.

In this paper we discuss the regioselectivity of the N-ethylation reaction of *N*-benzyl-4-oxo-1,4-dihydroquinoline-3-carboxamide (**5**). We correlate the experimental results with theoretical calculations, and with this we propose different hypotheses in the sense of better explaining the observed regioselectivity.

## Results and Discussion

### Synthesis

**Scheme 1:**
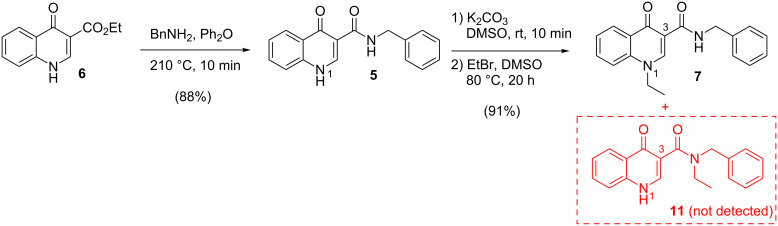
Synthetic route for the preparation of 1-ethyl-4-oxoquinoline-3-carboxamide **7**.

Intermediate **6** was synthesized through the Gould–Jacobs method [24–26] and was first subjected to the carbonyl nucleophilic substitution reaction with benzylamine, according to a procedure already described in the literature [15–16]. The isolated carboxamide **5** was then treated with potassium carbonate followed by a dropwise addition of bromoethane, as the alkylating agent. This synthetic strategy provided exclusively the 1-ethylated product **7** with a good overall yield (80%, Scheme 1).

**Scheme 2:**
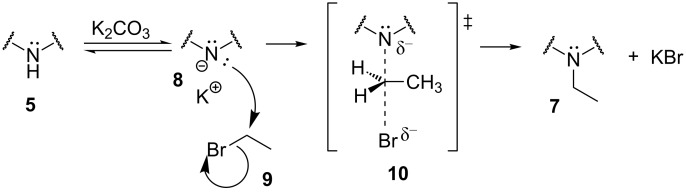
Reaction steps and main transition state leading to compound **7**.

Previous treatment of **5** with potassium carbonate promotes the establishment of an acid–base equilibrium, leading in situ to the formation of its conjugate base **8**. This anionic intermediate **8** acts as nucleophile and reacts with bromoethane (**9**) in a, probably, bimolecular mechanism, through a pentacoordinate transition state **10** as represented in Scheme 2.

The reaction occurs in a regioselective manner, without the formation of any byproduct derived from the N-alkylation of the amide group.

### Structural characterization

Table 1 gives the nuclear magnetic resonance spectroscopic data that allowed to confirm the structures of substances **5** and **7**, and thus also confirmed the regioselectivity of the alkylation reaction.

**Table 1:** ^1^H NMR (DMSO-*d*_6_, 500.00 MHz) data of compounds **5** and **7**, and the correlations observed from the ^1^H,^1^H-COSY and ^1^H,^13^C-HMBC spectra.^a^

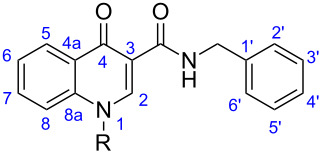						

#H	**5** (R = H)			**7** (R = Et)		

	δ (ppm) [m; *n*H; *J* (Hz)]	COSY correlations	HMBC correlations	δ (ppm) [m; *n*H; *J* (Hz)]	COSY correlations	HMBC correlations

1	ud	ud	–	–	–	–
2	8.78 [s; 1H]	ud	*C*ONH; C-4; C-3; C-8a	8.91 [s; 1H]	–	N*C*H_2_CH_3;_ C-8a; *C*ONH; C-4
5	8.26 [dd; 1H; 8.5 and 1.2]	H-6	C-4; C-7; C-8a	8.35 [dd; 1H; 7.9 and 1.8]	H-6	C-7; C-8a; C-4
6	7.48–7.44 [m; 1H]	H-5; H-7	C-4a; C-8; C-7	7.53 [t; 1H; 7.9]	H-5; H-7	C-8; C-4a; C-7
7	7.76–7.72 [m; 1H]	H-6	C-8a; C-5	7.86–7.81 [m; 1H]	H-6; H-8	C-5; C-8a
8	7.69 [d; 1H; 7.9]	*	C-4a; C-6	7.89 [d; 1H; 8.5]	H-7	C-6; C-4a; C-7
CON*H*	10.43 [t; 1H]	CONHC*H*_2_	*C*ONH; CONH*C*H_2_	10.37 [t; 1H; 5.5]	CONHC*H*_2_	CONH*C*H_2_
CONHC*H*_2_	4.58 [d; 2H; 6.1]	CON*H*	*C*ONH; C-1'; C-2'/6'	4.58 [d; 2H; 5.5]	CON*H*	*C*ONH; C-1'; C-2'/6'
2’/6’ and 3'/5'	7.38–7.31 [m; 4H]	H-4'	C-1'; C-4'	7.38–7.31 [m; 4H]	H-4'	*
4'	7.27–7.22 [m; 1H]	H-2'/6'; H-3'/5'	C-2'/6'; C-3'/5'	7.28–7.23 [m; 1H]	H-2'/6'; H-3'/5'	*
NCH_2_C*H*_3_	–	–	–	1.40 [t; 3H; 7.3]	NC*H*_2_CH_3_	N*C*H_2_CH_3_
NC*H*_2_CH_3_	–	–	–	4.51 [q; 2H; 7.3]	NCH_2_C*H*_3_	NCH_2_*C*H_3_; C-8a; C-2

^a^ud: undetected; *it was not possible to differentiate in the spectrum.

In the ^1^H NMR spectrum of derivative **5**, it was possible to readily assign the singlet at 8.78 ppm as related to the H-2 resonance. The signal of hydrogen H-1 was not observed in the spectrum. The double doublet at 8.26 ppm (*J* = 8.5 and 1.2 Hz) was attributed to the H-5 hydrogen, while hydrogen H-8 was assigned as the doublet signal at 7.69 ppm (*J* = 7.9 Hz).

Four sets of multiplets at 7.76–7.72 (1H), 7.48–7.44 (1H), 7.38–7.31 (4H) and 7.27–7.22 (1H) ppm were assigned to the H-7, H-6, H-2'/6'/3'/5' and H-4' resonances, respectively. Finally the triplet at 10.43 ppm and the doublet at 4.58 ppm, were related to CON*H* and CONHC*H*_2_ resonances, respectively.

As expected, the ^1^H NMR spectrum of derivative **7** has the same signal pattern as that observed in the spectrum of substance **5**.

The H-2 resonance was assigned as the singlet at 8.91 ppm. The signals at 8.35 ppm (dd, *J* = 7.9, and 1.8 Hz) and 7.89 ppm (d, *J* = 8.5 Hz) were assigned to H-5 and H-8 resonances, respectively. The triplet at 7.53 ppm (*J* = 7.9 Hz) was related to H-6 and three sets of multiplets at 7.86–7.81 (1H), 7.38–7.31 (4H) and 7.28–7.23 (1H) ppm were attributed to H-7, H-2’/3’/5’/6’ and H-4’, respectively. The presence of the *N*-ethyl group was confirmed by the quartet and the triplet at 4.51 ppm (*J* = 7.3 Hz) and 1.40 ppm (*J* = 7.3 Hz), related to methylene (NC*H*_2_CH_3_) and methyl (NCH_2_C*H*_3_) hydrogens, respectively. Also, as described for derivative **5**, the hydrogen of the (CON*H*) group appeared as a broad triplet at 10.37 ppm (*J* = 5.5 Hz) and the doublet at 4.58 ppm (*J* = 5.5 Hz) was assigned to the methylene hydrogens (CONHC*H*_2_).

**Figure 3:**
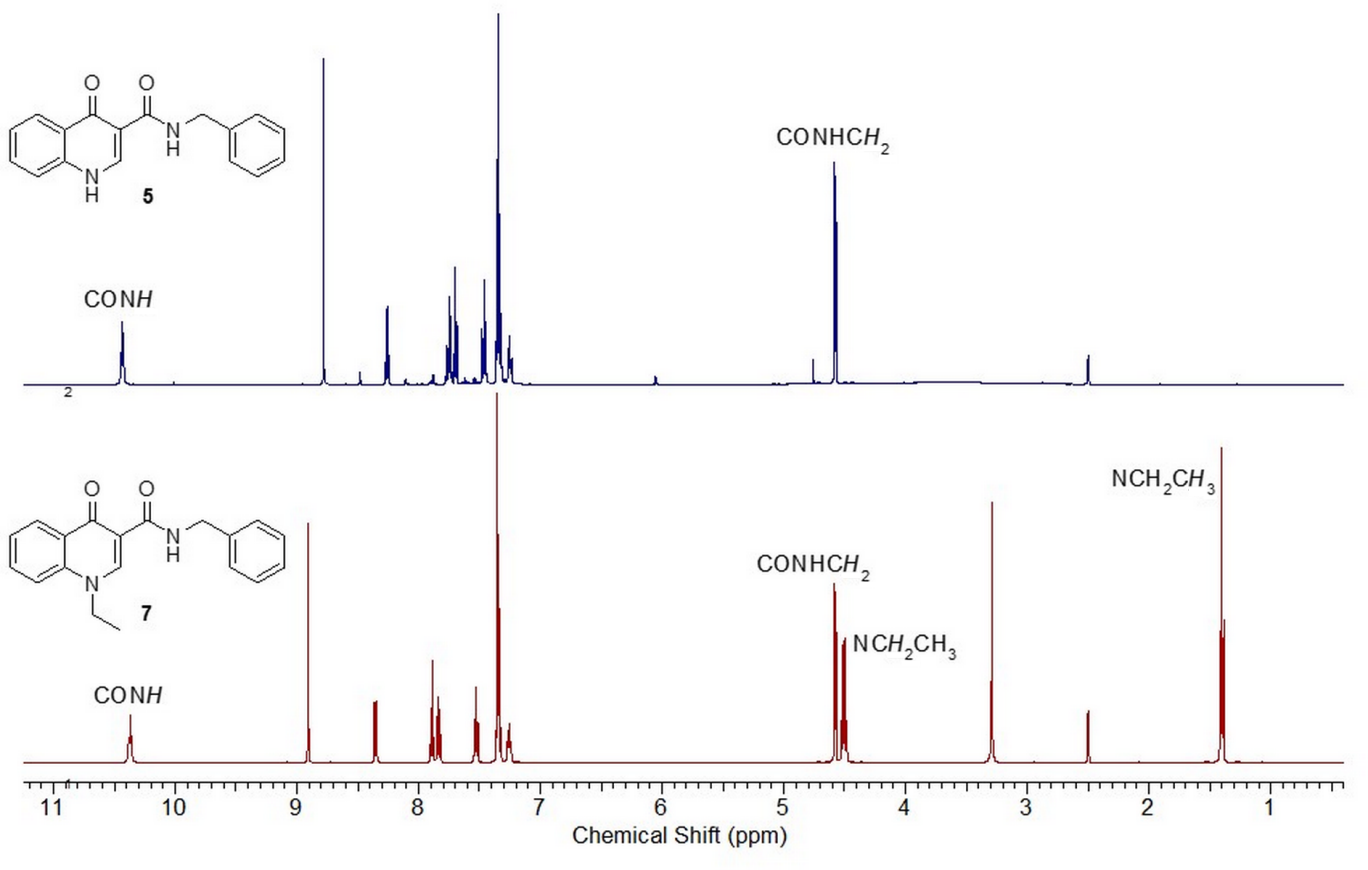
Same scale partial ^1^H NMR spectra of compounds **5** and **7** (DMSO-*d*_6_, 500 MHz).

A same scale comparison between the ^1^H NMR partial spectra for substances **5** and **7** is shown in Figure 3.

Derivatives **5** and **7** also had their structures confirmed by ^13^C-APT, COSY, HSQC and HMBC spectra.

**Figure 4:**
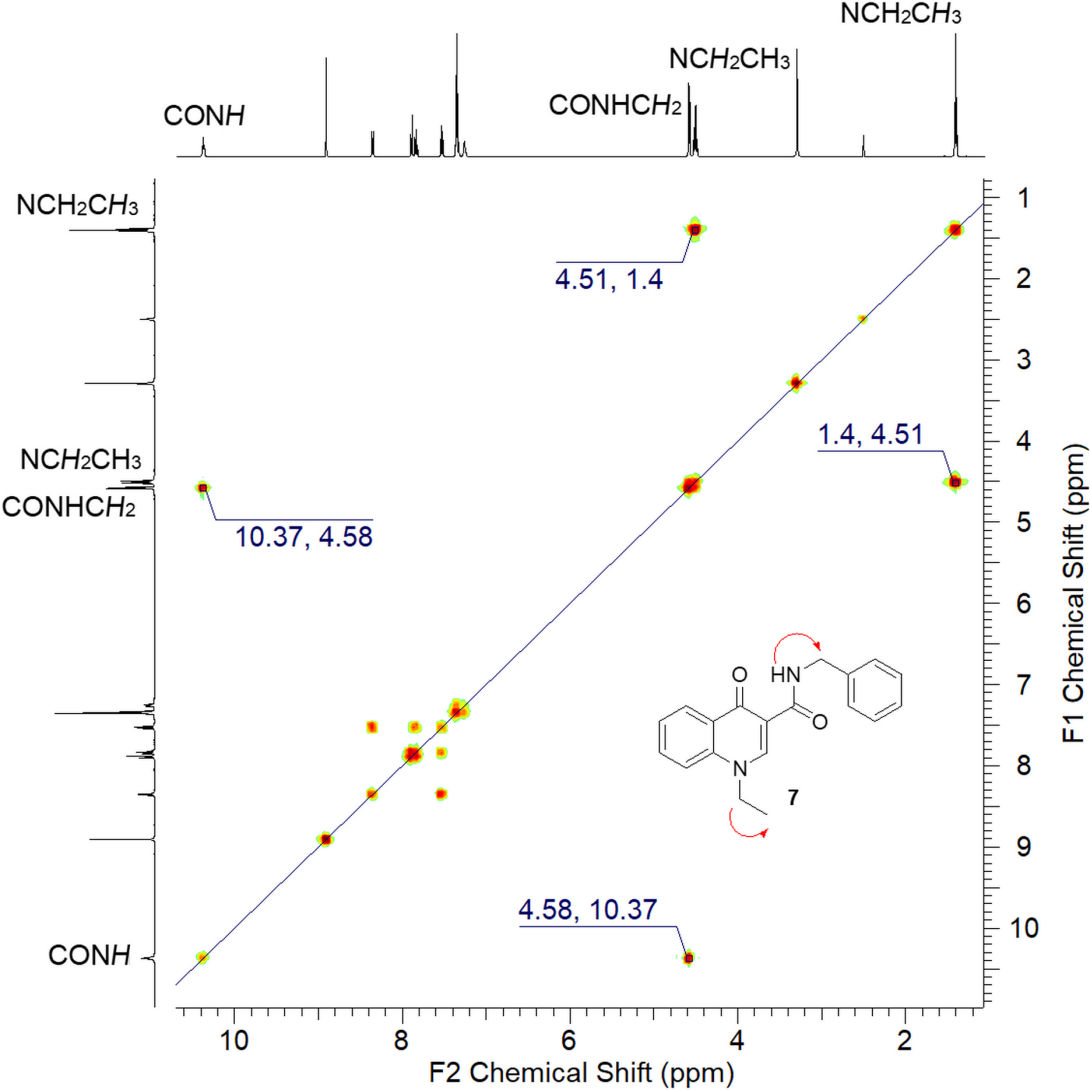
^1^H,^1^H-COSY spectrum of derivative **7** (DMSO-*d*_6_, 500 MHz).

In the COSY spectrum of **7**, the correlations between the hydrogen of the amide group (CONH) and of the benzylic hydrogens (CONHCH_2_Ph) confirm again the occurrence of the alkylation in the nitrogen of the oxoquinoline nucleus (Figure 4).

**Figure 5:**
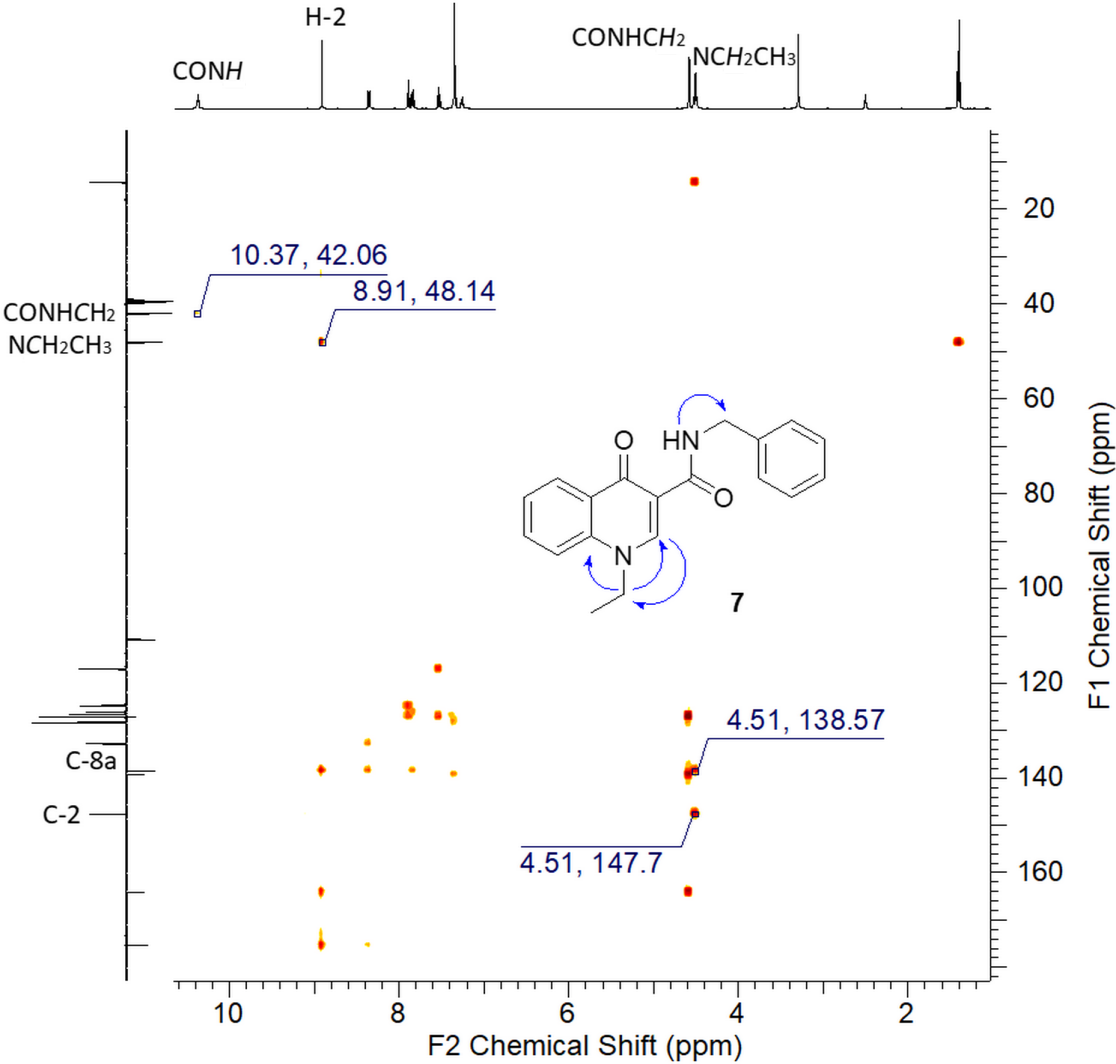
Partial HMBC spectrum of derivative **7** (DMSO-*d*_6_, 500 MHz).

From the HMBC spectrum of **7** it was observed that H-2 at δ = 8.91 ppm shows long range correlation with the methylenic carbon resonance for N*C*H_2_CH_3_ group at δ = 48.14 ppm (^3^*J*_CH_) and that the respective methylenic hydrogens at δ = 4.51 ppm are correlated with the carbon resonance for C8a at δ = 138.57 ppm (^3^*J*C_H_). CON*H* hydrogen signal at δ = 10.37 ppm is correlated with the carbon resonance for benzylic carbon at δ = 42.06 ppm (^2^*J*_CH_, Figure 5). These data also confirm the regioselectivity of the N-ethylation reaction.

**Figure 6:**
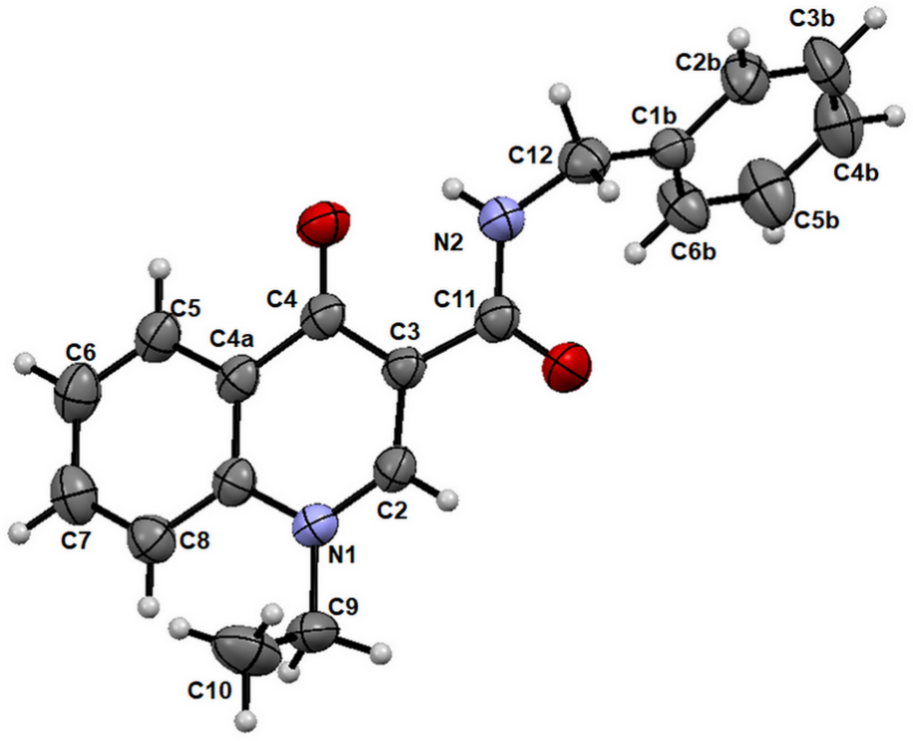
Asymmetric unit of product **7**.

Furthermore, adequate crystals of compound **7** were obtained from a mixture of ethanol and DMSO, which allowed the unambiguous resolution of its structure as shown in Figure 6.

As can be seen in Figure 6, attached to the central oxoquinoline ring is an ethyl substituent bonded to N1 and the carboxamide group is attached to C3. The solid-state structure confirms the regioselectivity of the reaction.

It is important to note from the crystalline structure that the intramolecular hydrogen bond between the hydrogen of the amide group, CON–H, and the carbonyl oxygen (C-4), promotes coplanarity between the oxoquinoline nucleus and the CONH moiety of this amide group connected to C-3, as expected.

An additional crystallographic description and the full one-dimension spectra of compound **7** are available in Supporting Information File 1.

### Theoretical data

Two main approaches were considered to better understand the reactivity of substrate **5** and, consequently, the regioselectivity of the reaction: quantification of the acidity of the N–H units and comparative analysis of the possible reaction paths.

#### Acidity of the N–H units

**Scheme 3:**
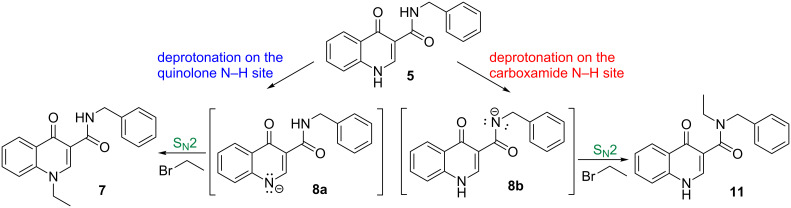
Deprotonation of **5** forming **8a** and **8b**, followed by reaction with bromoethane leading to products **7** and **11**.

A first possibility to rationalize the reactivity of carboxamide **5** was its deprotonation to produce a nucleophilic species which would then attack the bromoethane to produce the derivatives **7** or **11**. Therefore, we considered the deprotonation of both the oxoquinoline core and the carboxamide N–H sites, followed by the alkylation of the respective anion **8a** and **8b**, providing two possible products **7** and **11**, from which the results obtained could be compared (Scheme 3).

**Scheme 4:**
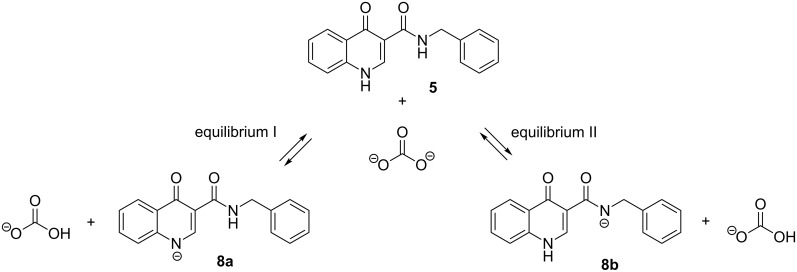
Acid–base equilibria considered for the data displayed in Table 2.

The acidity of both carboxamide and oxoquinoline N–H sites were compared in gas and condensed phase using water and DMSO. The solvent effects were included according to the polarized continuum solvation model (IEFPCM) [27–28]. The presence of the base used in the synthesis was also taken into consideration within both equilibria (Scheme 4 and Table 2).

**Table 2:** Main thermodynamic data obtained from the acid–base equilibria considered (kcal·mol^−1^).

	gas phase		H_2_O		DMSO	

	Δ*H*	Δ*G*	Δ*H*	Δ*G*	Δ*H*	Δ*G*

equilibrium I	−156.756	−154.456	−18.877	−19.458	−20.082	−21.349
equilibrium II	−115.211	−112.130	4.147	3.333	3.297	2.474

From these results, only equilibrium I, referring to the deprotonation of the oxoquinoline N–H presented negative values of Δ*H* and Δ*G* in all cases, characterizing the reaction as an exothermic one and favorable to the formation of the respective conjugate base, under such conditions. Deprotonation of the carboxamide hydrogen CON–H (equilibrium II), only presented negative values of Δ*H* and Δ*G* when considering gas phase and, even so, such values were not as significant as those from equilibrium I. Considering water and DMSO, the reaction becomes endergonic, suggesting that the carboxamide N–H site is not acidic enough for the deprotonation reaction using potassium carbonate as a base.

**Scheme 5:**
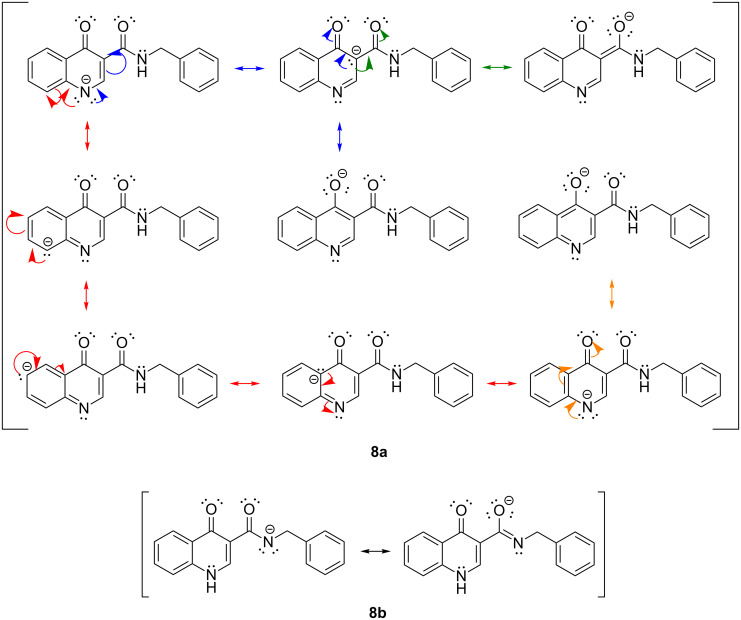
Charge dispersion due to resonance effects for both deprotonated species.

These results are in agreement with the analysis of the stability of the conjugate bases due to structural electronic effects. The oxoquinoline conjugate base presents a great stability, since it promotes a greater dispersion of the negative charge due to the two conjugated carbonyls (C-4 and CONH) and the adjacent aromatic system. At least nine main resonance structures involved in charge dispersion can be identified for this species. The carboxamide conjugate base, on the other hand, has its charge dispersed by only two main resonance structures due to the adjacent carbonyl, being therefore, less stable (Scheme 5).

#### Reaction paths analysis

The paths for the S_N_2 N-alkylation reaction of both deprotonated species (oxoquinoline and carboxamide N–H units) using bromoethane were obtained. It is worth highlighting that for each of the media considered, the N–C–Br bond angle (α) on the transition states were slightly higher for the carboxamide group (Table 3).

**Table 3:** Activation energies and enthalpies of both oxoquinoline and carboxamide N-ethylation reaction in different media (kcal·mol^−1^).

	gas phase			H_2_O			DMSO		

	α^a^	*E_a_*	Δ*H*	α^a^	*E_a_*	Δ*H*	α^a^	*E_a_*	Δ*H*

oxoquinoline	159.82°	14.6	−7.3	160.57°	11.0	−25.4	160.60°	11.4	−24.7
carboxamide	162.59°	9.8	−19.9	162.37°	9.5	−31.8	162.44°	9.5	−31.7

^a^N–C–Br bond angle at the transition state.

These results are consistent with the one previously found. As already shown, the oxoquinoline conjugate base is more stabilized when compared to the carboxamide one. Such verification is, for example, justified by the analysis of the electronic effects, as shown in the Scheme 5. Precisely because it is more unstable, the carboxamide conjugate base is a more reactive nucleophile and, therefore, associated with a lower energy barrier for the nucleophilic substitution reaction. Table 4 illustrates the optimized geometry for the transition states for each possible reaction path.

**Table 4:** Transition states associated with both reaction paths.

	oxoquinoline N-ethylation	carboxamide N-ethylation

transition state representation	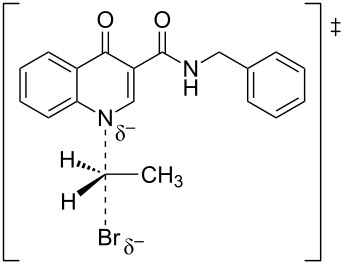	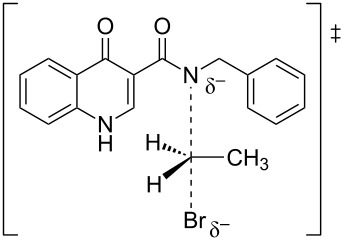
gas phase	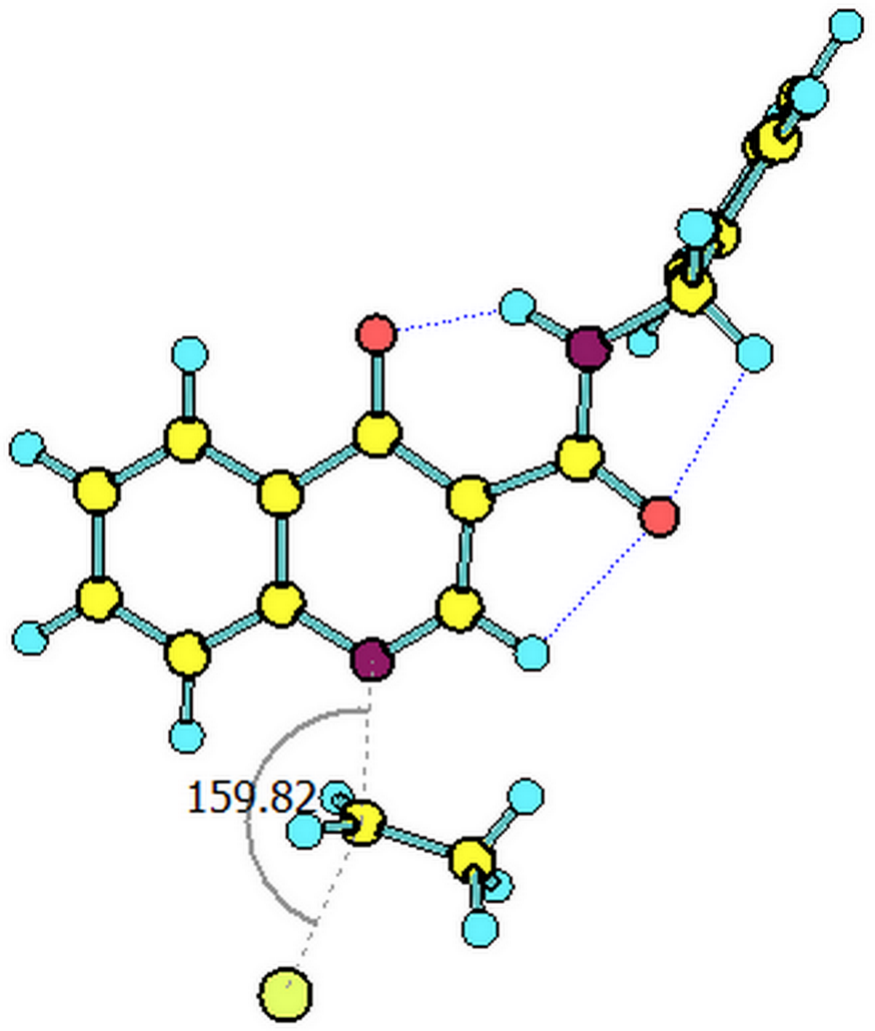	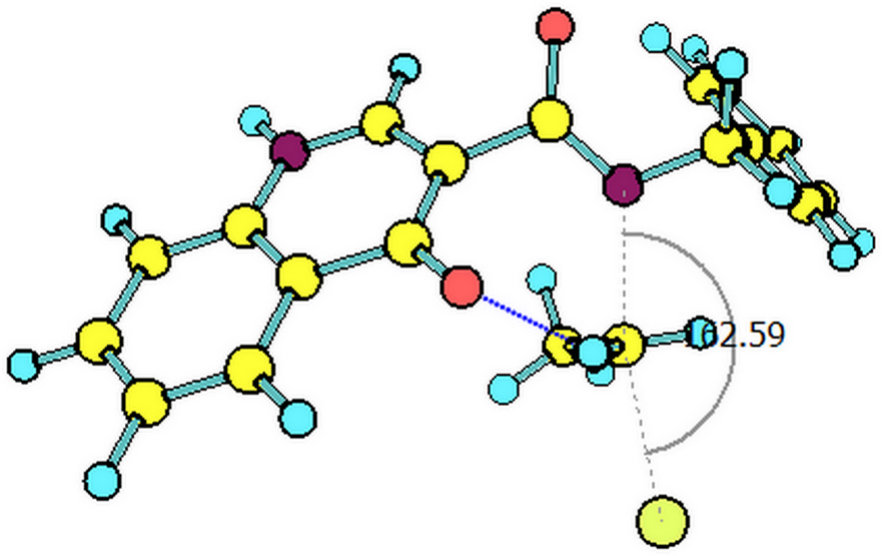
H_2_O	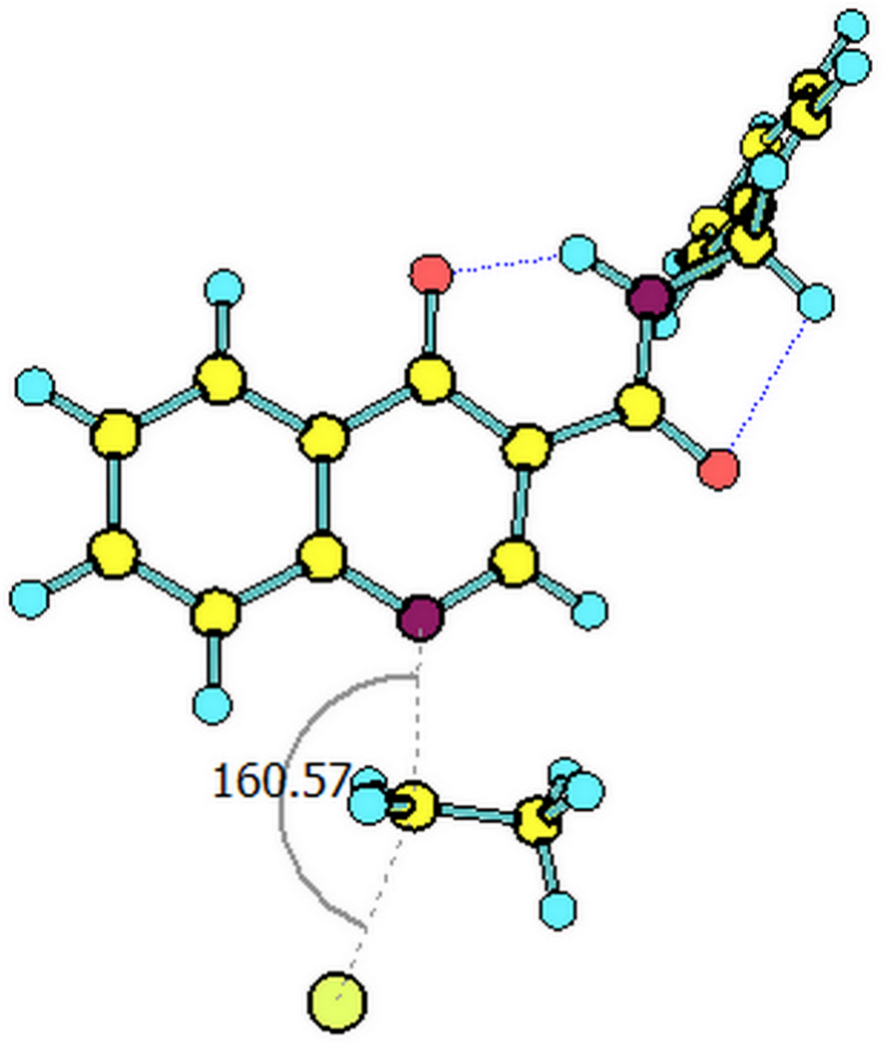	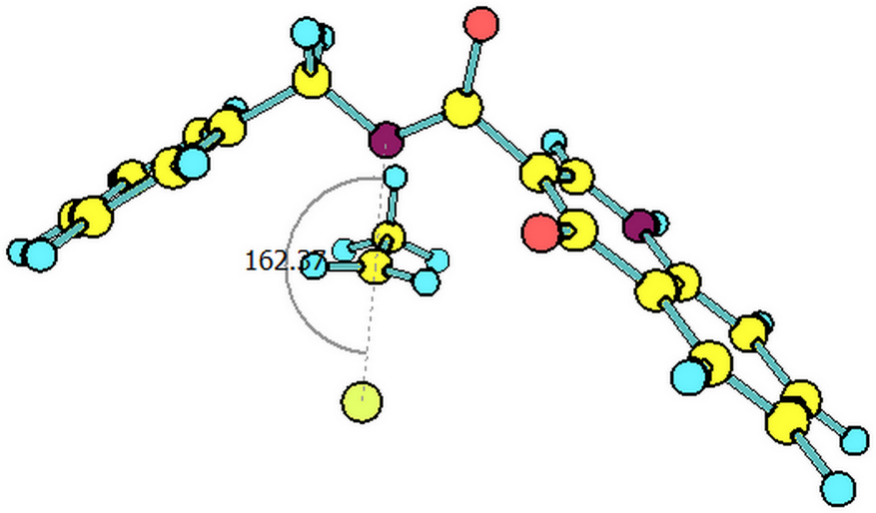
DMSO	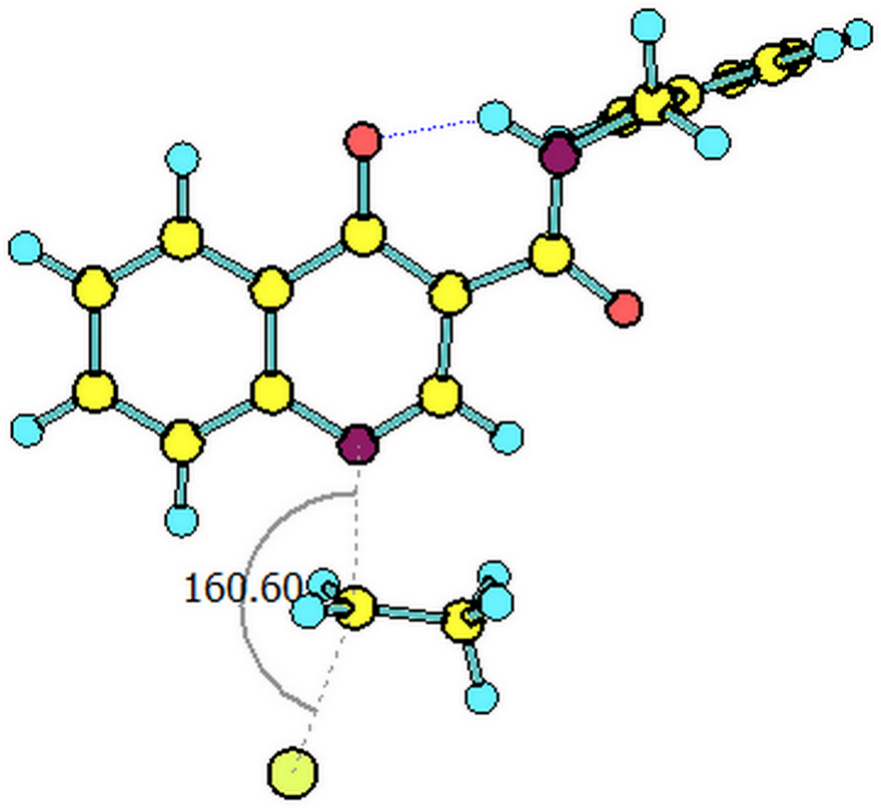	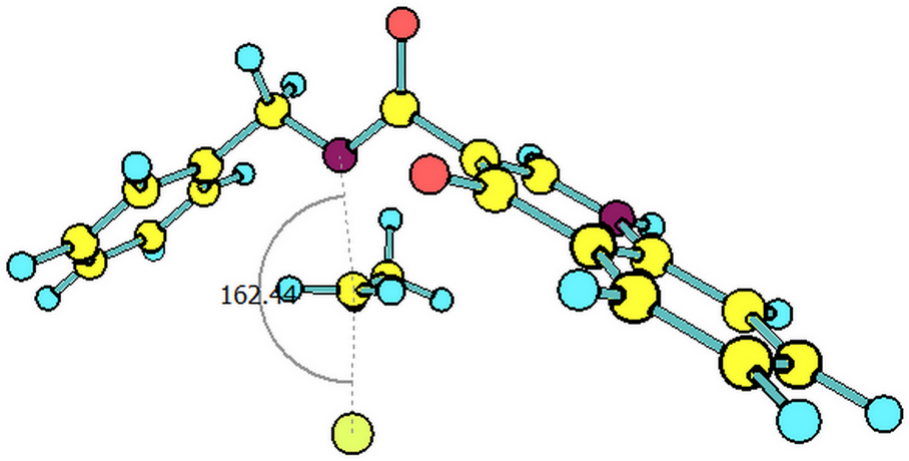

The comparison of the two possible reaction paths shows that although N-ethylation of the carboxamide site is associated with a lower energy barrier, addition of solvent stabilizes the transition state of the reaction through the oxoquinoline anion more than that corresponding to the carboxamide. Again the difference between the solvents is insignificant. It is consistent to deduce that a conjugate base resulting from deprotonation of the carboxamide would be much more nucleophilic than that of oxoquinoline, since from the acidity predictions, the oxoquinoline conjugate base is much less energetic than that of the carboxamide unit. If the latter species were also formed in the reaction medium, it would probably react faster and more exothermically than the nucleophile from the oxoquinoline nucleus. Since experimentally, this is not observed, it can be concluded that only the N–H site of oxoquinoline undergoes deprotonation. That is, the conjugate base of the carboxamide is not generated in the process.

Observing the predicted N–C–Br (α) angle in the calculated transition states, none of the optimized geometries provided the expected 180° angle for nucleophilic bimolecular substitution. In this regard, the transition state of the reaction with the carboxamide conjugate base provided a value of the N–C–Br bond angle about 2^o^ greater than that of the reaction with the oxoquinoline anion, in all cases considered. There was no significant difference for the N–C–Br angles when polar solvents were considered implicitly, however, it is noteworthy that a small approximation toward the 180° bond angle was observed for the transition state involving the nucleophile coming from of oxoquinoline.

These results indicate that the regioselectivity observed must occur due to the thermodynamics on the deprotonation step, leading exclusively to the oxoquinoline conjugate base as the reactive nucleophile.

## Conclusion

In conclusion, we studied the regioselectivity of the N-ethylation reaction of *N*-benzyl-4-oxo-1,4-dihydroquinoline-3-carboxamide (**5**). Three approaches were explored in order to understand the experimental results. The deprotonation of the N–H sites and the analysis of molecular orbitals corroborate the hypothesis that regioselectivity is a result of the higher acidity of the N–H hydrogen of the oxoquinoline, when compared to that of the carboxamide group. Because of this, the treatment with the base produces the reactive intermediate, the conjugate base resulting from the deprotonation of the oxoquinoline core, probably exclusively, which acts as the nucleophile in the S_N_2 reaction with bromoethane. These results are in agreement with the analysis of the reaction pathways from which we concluded that if the conjugate base of the carboxamide group were formed in the reaction medium, the S_N_2 reaction would most likely occur in this site, since this reaction would be the kinetic and thermodynamically most favored one.

## Experimental

### General

All reagents and solvents were purchased from Merck & Co (Kenilworth, New Jersey, USA) and used without further purification. Melting points were measured with a Fisher–Johns apparatus. NMR spectra were recorded on a Varian spectrometer operating at 500 MHz (^1^H) and 125 MHz (^13^C), using DMSO-*d*_6_ as the solvent. Chemical shifts were reported in parts per million (ppm) relative to the internal standard tetramethylsilane (TMS). Hydrogen and carbon NMR spectra were typically obtained at room temperature. The two-dimensional experiments were conducted using standard Varian Associates automated programs for data acquisition and processing. Both the starting material **5** [16] and the ethylated product **7** [29] have already been described in the literature. Substance **5** was synthesized through a known procedure [15–16]. The ethylation procedure is described below.

#### Procedure for the preparation of *N*-benzyl-1-ethyl-4-oxo-1,4-dihydroquinoline-3-carboxamide (**7**)

In a round bottom flask, 1.0 g (3,6 mmol) of *N*-benzyl-4-oxo-1,4-dihydroquinoline-3-carboxamide (**5**), 1.4 g (10.1 mmol) of potassium carbonate and 10.0 mL of dimethyl sulfoxide (DMSO) were added and stirred at room temperature for 15 minutes. 1 mL (13.4 mmol) of bromoethane was added and the mixture was kept under 80 °C for 24 hours. The system was allowed to reach room temperature and the mixture was poured in ice and water. The solid was filtered and washed with water. No further purification process was necessary. Compound **7** was obtained as a white solid in 91% yield; mp 136–137 °C; ^1^H NMR (500 MHz, DMSO-*d*_6_) δ 10.37 (t, *J* = 5.5 Hz, 1H, CON*H*), 8.91 (s, 1H, H-2), 8.35 (dd, *J* = 7.9 and 1.8 Hz, 1H, H-5), 7.89 (d, *J* = 7.9 Hz, 1H, H-8), 7.86–7.81 (m, 1H, H-7), 7.53 (t, *J* = 7.9 Hz, 1H, H-6), 7.38–7.31 (m, 4H, H-2’/H-6’ and H-3’/H-5’), 7.28–7.22 (m, 1H, H-4’), 4.58 (d, *J* = 5.5 Hz, 2H, CONHC*H*_2_), 4.51 (q, *J* = 7.3 Hz, 2H, NC*H*_2_CH_3_), 1.40 (t, *J* = 7.3 Hz, 3H, NCH_2_C*H*_3_); ^13^C NMR (125 MHz, DMSO-*d*_6_) δ 175.37 (C-4), 164.10 (*C*ONH), 147.70 (C-2), 139.36 (C-1'), 138.57 (C-8a), 132.87 (C-7), 128.28 (C-3'/5' or C-2’/6’), 127.21 (C-2'/6' or C-3’/5’), 127.19 (C-4a), 126.72 (C-4’), 126.17 (C-5), 124.83 (C-6), 117.12 (C-8), 110.87 (C-3), 48.14 (N*C*H_2_CH_3_), 42.06 (CONH*C*H_2_), 14.33 (NCH_2_*C*H_3_).

#### X-ray diffraction measurement

Single crystal X-ray diffraction data of derivative **7** were collected on a Bruker D8 Venture diffractometer at room temperature, using a microfocus X-ray source using Mo Kα radiation (λ = 0.71073 Å). The crystal was mounted on a Kappa goniometer. Reflections were collected at room temperature, using a PHOTON 100 detector, which uses a CMOS sensor. Data collection and cell refinement were performed with the Bruker Instrument Service APEX2 v4.2.2 [30], and the data integration was carried out using SAINT [31]. Empirical multi-scan absorption correction, using equivalent reflections, was performed with the SADABS program [32]. The structure solutions, using direct methods, were performed with the SHELXS-2013. The Full-matrix least-squares refinements based on F2 were performed with the SHELXL-2013 program packages [33] using the WinGX software interface [34]. Anisotropic parameters were refined to all non-hydrogen atoms. Hydrogen atoms positions were constrained to neutral diffraction distances values [35]. The crystallographic table was mounted using the OLEX2 software [36].

#### Computational details

All the calculations were carried out with the Gaussian 09 software package [37] considering the absence (gas phase) and the presence of two implicit solvents (water and DMSO), using the polarized continuum solvation model (IEFPCM) [27–28]. All computations were done with the DFT functional B3LYP [28,38–41] and the 6-31+G(d) basis set [42–43], as implemented in the Gaussian 09 suit of programs. All geometries were fully optimized and then characterized as minima on the potential energy surface (no negative eigenvalue in the Hessian second order matrix) [28,38–41].

## Supporting Information

File 1 X-ray crystallographic data and copies of NMR spectra for compound **7**.
