# Supplementary material for: Study on the regioselectivity of the N-ethylation reaction of N-benzyl-4-oxo-1,4-dihydroquinoline-3-carboxamide
Source: Beilstein J Org Chem. 2019 Feb 12;15:388–400. doi: 10.3762/bjoc.15.35 (PMC6404479; doi:10.3762/bjoc.15.35)
Supplement: File 1 — X-ray crystallographic data and copies of NMR spectra for compound 7. [file Beilstein_J_Org_Chem-15-388-s001.pdf]

BEILSTEIN JOURNAL OF ORGANIC CHEMISTRY

Supporting Information

for

Study on the regioselectivity of the N-ethylation reaction of *N*-benzyl-4-oxo-1,4-dihydroquinoline-3-carboxamide

Pedro N. Batalha, Luana da S. M. Forezi, Maria Clara R. Freitas, Nathalia M. de C. Tolentino, Ednilsom Orestes, José Walkimar de M. Carneiro, Fernanda da C. S. Boechat and Maria Cecília B. V. de Souza

Beilstein J. Org. Chem. **2019,** *15,* 388–400. doi:10.3762/bjoc.15.35

X-ray crystallographic data and copies of NMR spectra for compound 7

License and Terms: This is a supporting information file under the terms of the Creative Commons Attribution License (http://creativecommons.org/licenses/by/4.0). Please note that the reuse, redistribution and reproduction in particular requires that the authors and source are credited.
The license is subject to the *Beilstein Journal of Organic Chemistry* terms and conditions: (https://www.beilstein-journals.org/bjoc)

Single crystal description

A single crystal from substance **7** was obtain and analyzed by XRD for structure determination.

The designed compound **7** crystalized in the monoclinic system with a centrosymmetric space group, *P*21/*c*. Crystallographic data, as cell parameters, and refinement statistical indicators within some molecular information are present in Table S1 for the refined model.

Table S1: Crystal data and structure refinement for compound **7**.

Empirical formula	$C_{19}H_{18}N_2O_2$
Formula weight	306.35
Temperature (K)	293(2)
Crystal system	monoclinic
Space group	P21/c
A (Å)	12.0869(13)
B (Å)	23.193(3)
C (Å)	5.7302(7)
Α	90 °
β	102.475(4) °
γ	90 °
Volume (Å^3)	1568.4(3)
Z	4
P_{calcg} (cm^3)	1.297
M (mm^{-1})	0.085
Crystal size (mm^3)	0.778 × 0.287 × 0.206
Radiation	MoKα (λ = 0.71073)
2Θ range for data collection	4.924 ° to 52.87 °
Index ranges	-15 ≤ h ≤ 15, -29 ≤ k ≤ 29, -7 ≤ l ≤ 7
Reflections collected	23284
Independent reflections	3151 [R_{int} = 0.1239, R_σ = 0.0628]
Data/restraints/parameters	3151/0/209
Goodness-of-fit on F2	1.102
Final R indexes [I>=2σ (I)]	R1 = 0.0657, wR2 = 0.1224
Final R indexes [all data]	R1 = 0.1215, wR2 = 0.1436
Largest diff. peak/hole / e Å-3	0.17/-0.20

The asymmetric unit corresponds to **7** and is shown in **Figure S1**. There are four molecules per unit cell.

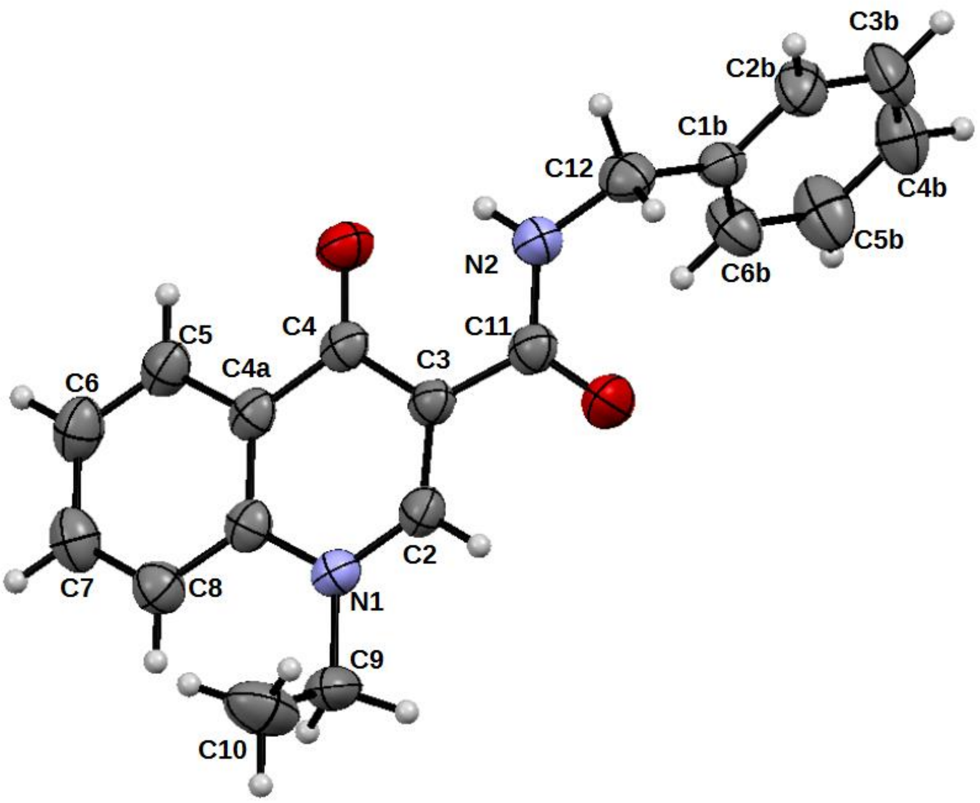

Figure S1: Asymmetric unit of product **7**.

As can be seen in Figure S1, attached to the N1 of the central oxoquinoline ring is the ethyl group, and in C3 the carboxamide group. The solid-state structure confirms the regioselectivity of the reaction.

The central ring, as expected, displays a planar geometry with r.m.s. deviation equal to 0.0153. The C–C average distance are equal to 1.400 Å. (Table S2), in the range of double and single C–C bonds. Both geometrical parameters are in accordance to an aromatic system. Those geometrical parameters are in agreement with the NMR analysis, in which the chemical shifts for the hydrogens attached to the oxoquinoline core are assigned in the aromatic chemical shift region.

The aliphatic C–C bond distances are in the range of simple bonds. The NMR chemical shifts were also in agreement with this.

Table S2: Bond Lengths for compound **7**.

**Bond**	**Bond Length (Å)**
O1-C4	1.254(3)
O2-C11	1.235(3)
N1-C2	1.344(3)
N1-C8a	1.398(3)
N1-C9	1.473(3)
N2-C11	1.338(3)
N2-C12	1.442(3)
C1b-C6b	1.376(3)
C1b-C2b	1.382(3)
C1b-C12	1.505(3)
C4a-C8a	1.406(3)
C4a-C4	1.459(3)
C4a-C5	1.400(3)
C11-C3	1.482(3)
C2-C3	1.370(3)
C8a-C8	1.401(3)
C4-C3	1.439(3)
C6b-C5b	1.381(4)
C2b-C3b	1.375(4)
C5-C6	1.362(4)
C8-C7	1.366(4)
C9-C10	1.510(4)
C6-C7	1.393(4)
C3b-C4b	1.364(4)
C5b-C4b	1.361(4)

In solid state the molecule conformation can be defined by three planes, as can be seen in Figure S1. The first plane comprehends the oxoquinoline ring which is coplanar to

C11 and N2. This coplanarity is caused due intramolecular H-bond restriction between O1 acceptor and N2–H2, with the formation of a six membered ring. As described before, this intramolecular H-bond is of great importance for biological properties.

The second plane is defined by the carboxamide group and the phenyl ring. This last one is almost perpendicular to the oxoquinoline, forming an angle equal to 84.49(8)°. The first observation that could lead to the phenyl orientation is the presence of a medium intramolecular H-bond, formed between H6b and N2. This causes the phenyl orientation in space. This H-bond could cause chemical shift differentiation for this H atom. However as expected in solution the molecular conformation is not restricted to the one observed in the solid packing, and as consequence the NMR analysis could not differentiate H2b and H6b, being them chemically equivalent in this condition.

Another feature that could cause the phenyl orientation is the supramolecular array. C–H/π intermolecular interaction formed by the ethyl group and the phenyl ring are present, combined with a weak nonconventional H-bond formed by adjacent oxoquinoline C1–H1 and the O2 of the carboxamide group, which pulls over the phenyl moiety off the oxoquinoline ring plane. The third plane contains the ethyl group and the angle within the oxoquinoline plane is equal to 78.0(0.2)°.

Table S3: Main hydrogen bonds observed (H-bond distance lower than 2.000 Å and D–H/A angle higher than 110°).

D[a]-H bond	A[b]	D-H bond lenght (Å)	H/A distance (Å)	D-H/A angle (^{o})	D/A distance (Å)
N2-H2	O1	0.860	1.979	137.92	2.680
C2-H1	O2	0.930	2.355	157.17	3.232
C9-H9B	O2	0.970	2.450	146.49	3.302

a. D = Hydrogen bond donor atom

b. A = Hydrogen bond acceptor atom

NMR spectra

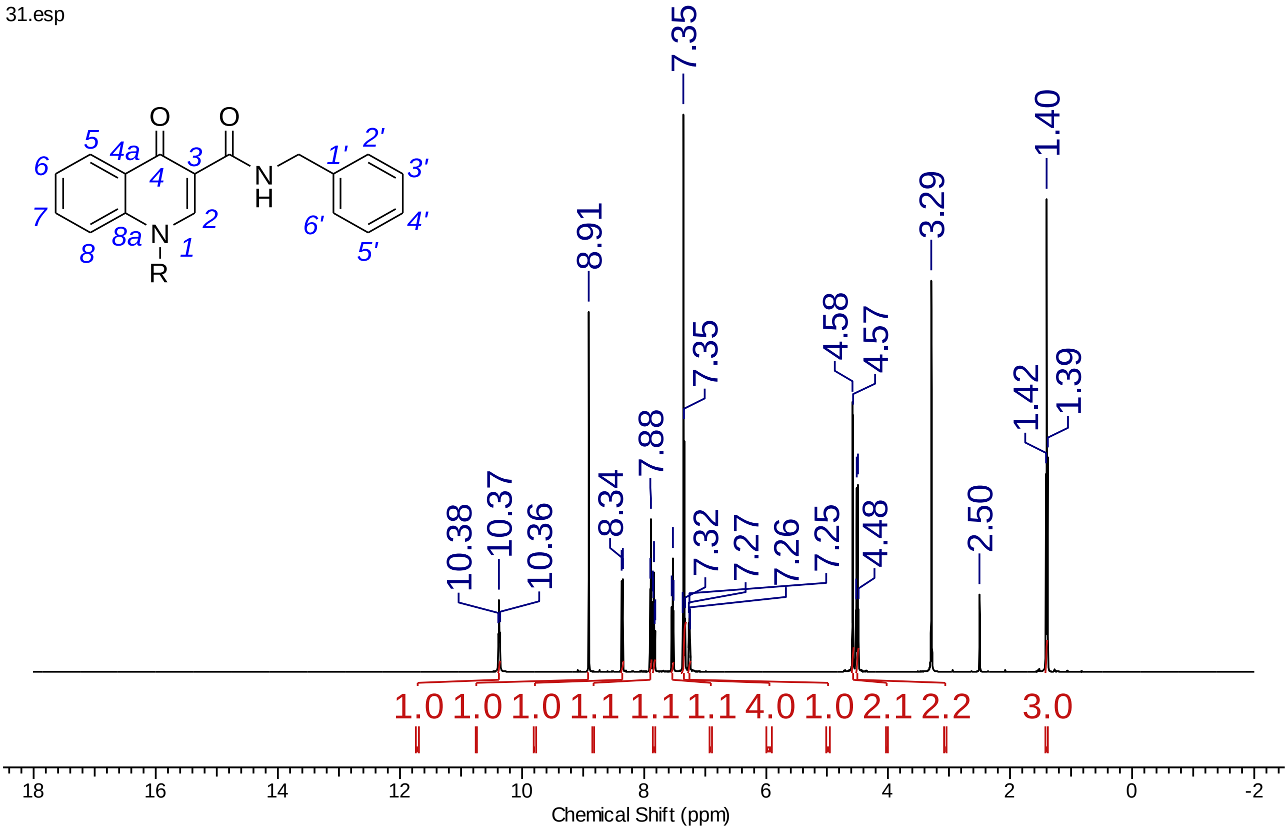

^{1}H NMR spectrum of derivative 7 (DMSO-d_6, 500 MHz).

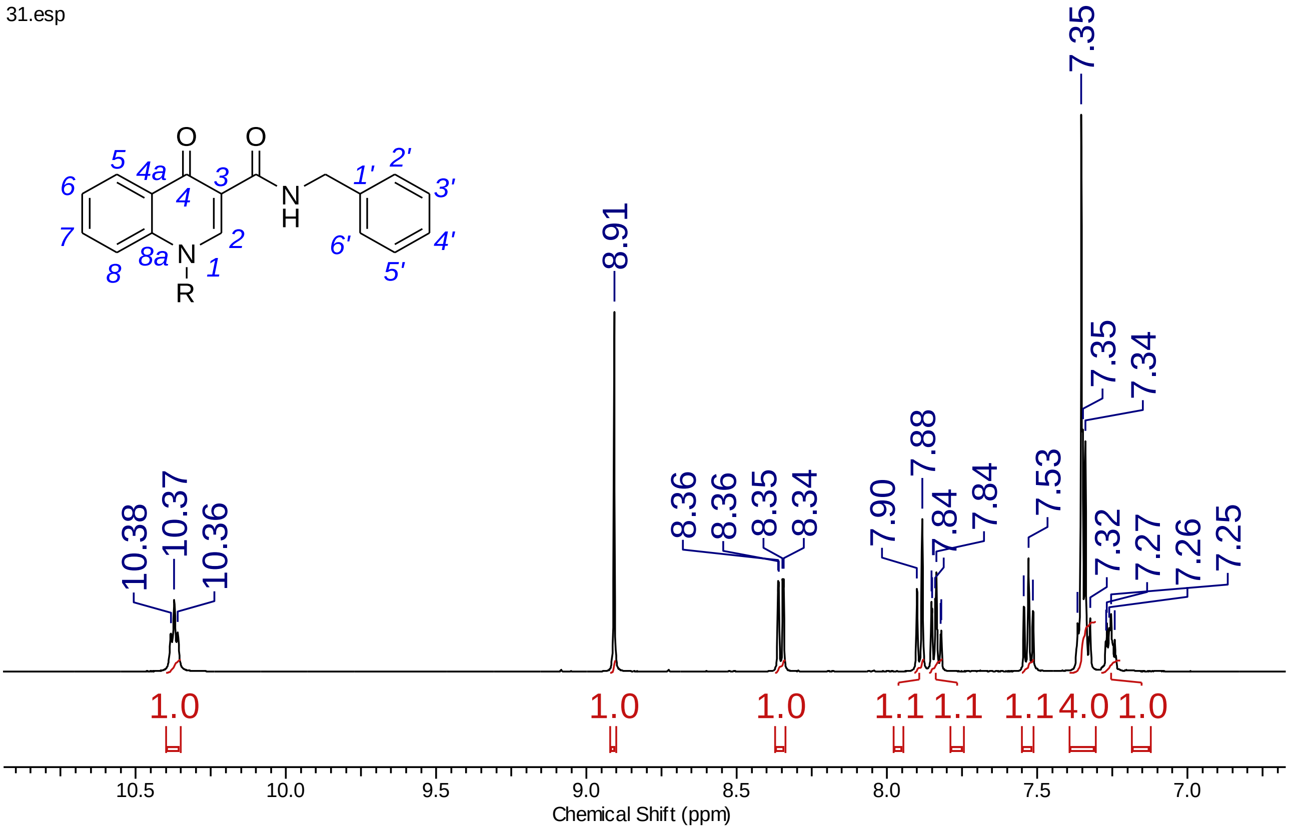

Expansion of the ^{1}H NMR spectrum of derivative 7 (DMSO-d_6, 500 MHz).

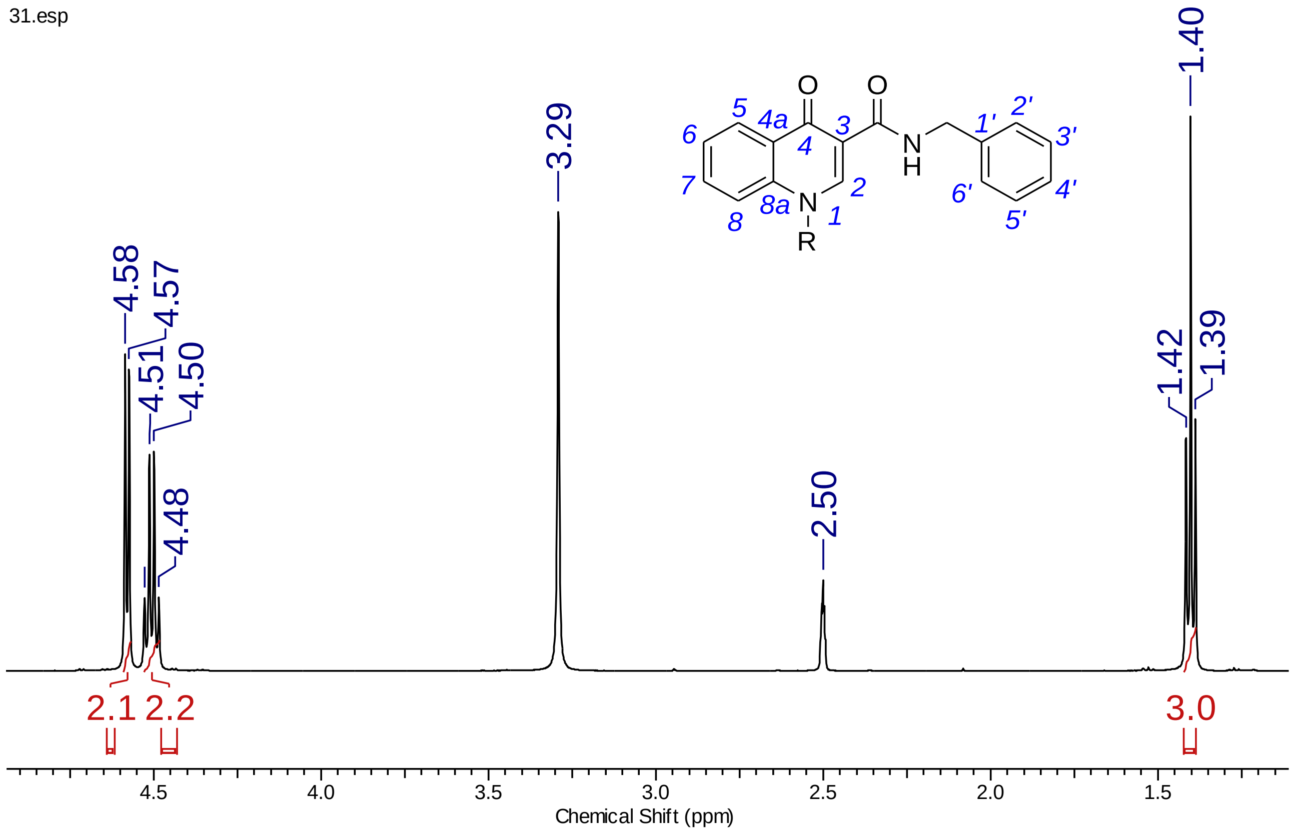

Expansion of the ¹H NMR spectrum of derivative 7 (DMSO-d_6, 500 MHz).

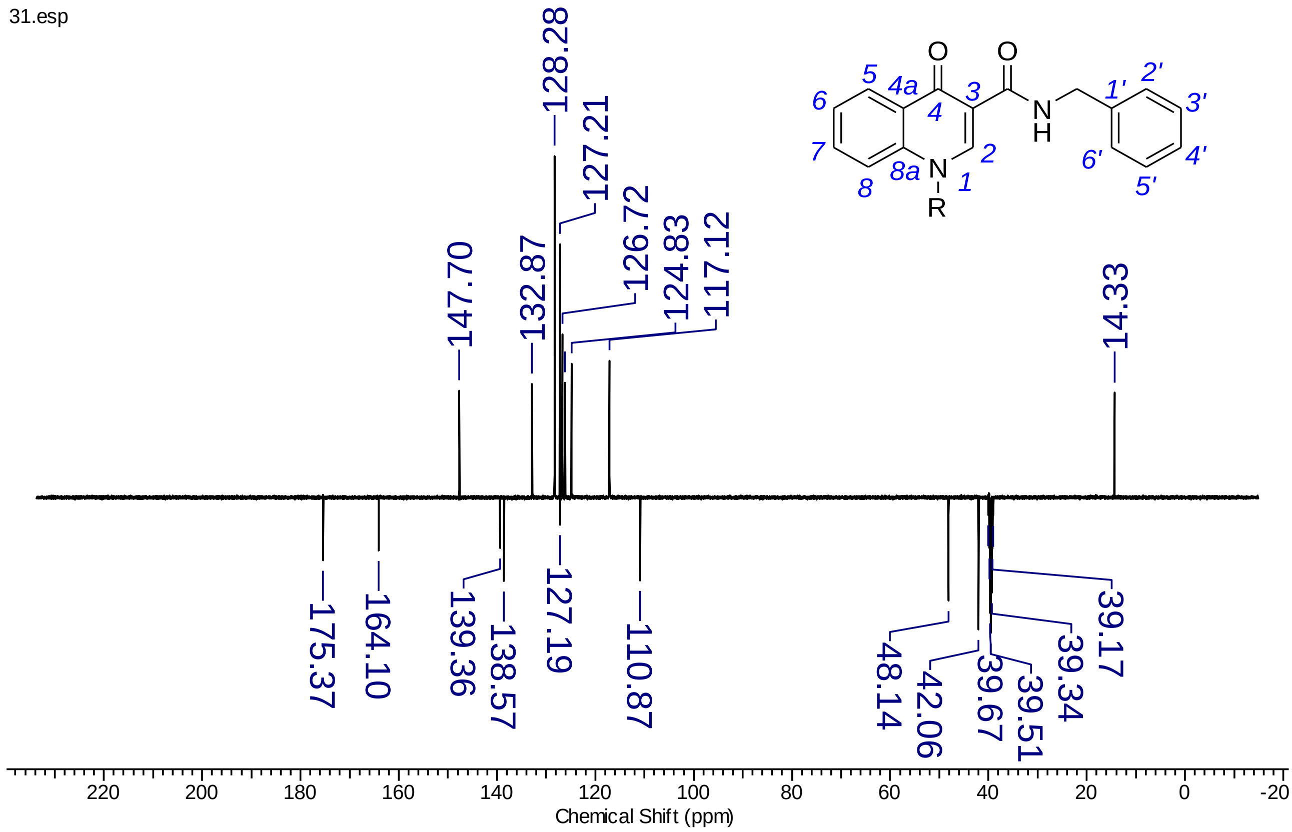

¹³C-APT NMR spectrum of derivative 7 (DMSO-d_6, 125 MHz).

31.esp

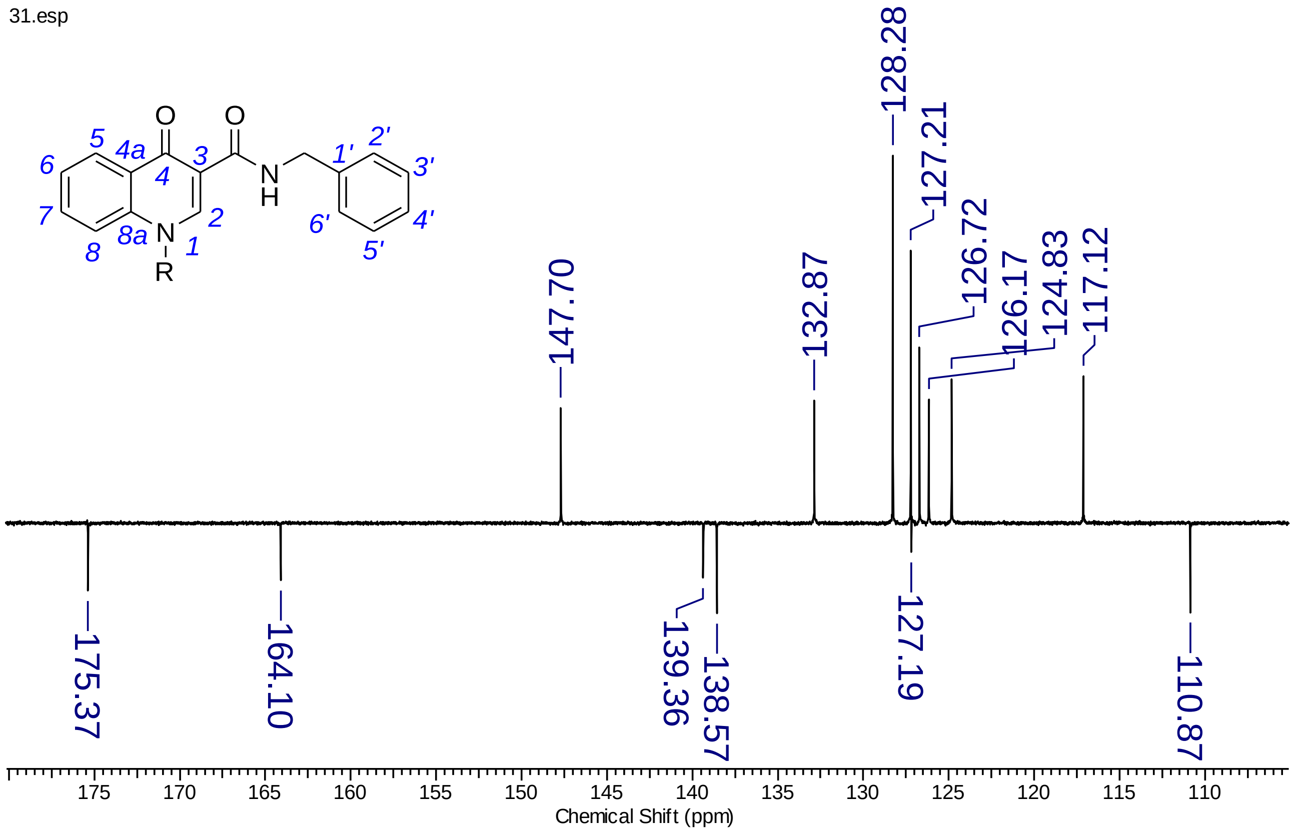

Expansion of the ^{13}C-APT NMR spectrum of derivative 7 (DMSO-d_6, 125 MHz).

31.esp

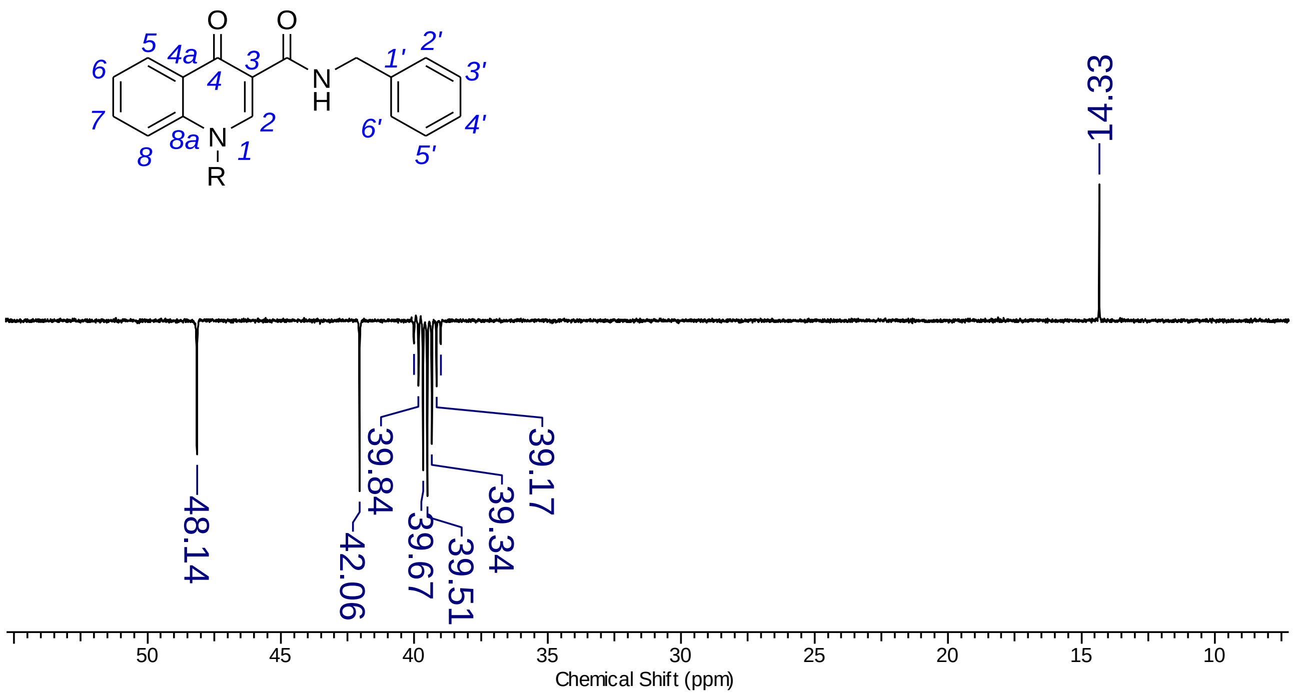

Expansion of the ^{13}C-APT NMR spectrum of derivative 7 (DMSO-d6, 125.00 MHz).
